# Transcriptome analysis of aphids exposed to glandular trichomes in tomato reveals stress and starvation related responses

**DOI:** 10.1038/s41598-022-24490-1

**Published:** 2022-11-23

**Authors:** Rosario Planelló, Lola Llorente, Óscar Herrero, Marta Novo, Lidia Blanco-Sánchez, Juan Antonio Díaz-Pendón, Rafael Fernández-Muñoz, Victoria Ferrero, Eduardo de la Peña

**Affiliations:** 1 Biology and Environmental Toxicology Group, Faculty of Science, Universidad Nacional de Educación a Distancia (UNED), 28232 Las Rozas, Madrid, Spain; 2 Biodiversity, Ecology and Evolution Department, Complutense University of Madrid, Jose Antonio Novais, 12, 28040 Madrid, Spain; 3 Institute for Subtropical and Mediterranean Horticulture (IHSM-UMA-CSIC), Spanish National Research Council (CSIC), Finca Experimental La Mayora, Algarrobo-Costa, 29750 Malaga, Spain; 4 Area of Botany, Faculty of Biological and Environmental Sciences, University of Leon, 24071 León, Spain; 5 Department of Plants and Crops, Faculty of Bioscience Engineering, Ghent University, Coupure Links 653, 9000 Ghent, Belgium

**Keywords:** Transcriptomics, Plant sciences, Secondary metabolism, Molecular biology, Transcription

## Abstract

Understanding the responses of insect herbivores to plant chemical defences is pivotal for the management of crops and pests. However, the mechanisms of interaction are not entirely understood. In this study, we compared the whole transcriptome gene expression of the aphid *Macrosiphum euphorbiae* grown on two different varieties of tomato that differ in their inducible chemical defences. We used two isogenic lines of tomato with a shared genetic background that only differ in the presence of type IV glandular trichomes and their associated acylsucrose excretions. This works also reports a de novo transcriptome of the aphid *M. euphorbiae*. Subsequently, we identified a unique and distinct gene expression profile for the first time corresponding to aphid´s exposure to type IV glandular trichomes and acylsugars. The analysis of the aphid transcriptome shows that tomato glandular trichomes and their associated secretions are highly efficient in triggering stress-related responses in the aphid, and demonstrating that their role in plant defence goes beyond the physical impediment of herbivore activity. Some of the differentially expressed genes were associated with carbohydrate, lipid and xenobiotic metabolisms, immune system, oxidative stress response and hormone biosynthesis pathways. Also, the observed responses are compatible with a starvation syndrome. The transcriptome analysis puts forward a wide range of genes involved in the synthesis and regulation of detoxification enzymes that reveal important underlying mechanisms in the interaction of the aphid with its host plant and provides a valuable genomic resource for future study of biological processes at the molecular level using this aphid.

## Introduction

Although insect herbivores potentially have an abundance of plant species available for feeding, herbivory is often limited by the defence mechanisms that plants have evolved to level off insect attacks^1^. The co-evolution of phytophagous insects and their host plants has shaped the evolutionary history of both groups^2,3^. Despite the importance of this 'arms race', our knowledge of the mechanisms operating is still limited, and while plant defences and their effects on herbivores are well documented, much less is known on the mechanisms evolved by insects to overcome these defences^4,5^.

Aphids are important pests on virtually all crops and are specialised herbivores that feed on the phloem of vascular plants^6^. They can greatly reduce crop yield by removing large quantities of sugar-rich sap from their hosts and by transmitting detrimental plant viruses and pathogens^7,8^. Compared to chewing insects, aphid feeding causes little structural harm to a plant. Negative impacts of sustained aphid feeding often arise from the rapid clonal reproduction of aphids and subsequent depletion of the plant's resources. Moreover, aphids can modulate and suppress the phytohormonal and defensive response^9,10^.

Plants fight the herbivores through direct defences affecting host plant preference or survival and reproductive success, and indirectly through other species such as natural enemies of the insect^11–13^. Direct defences are mediated by plant traits that confer mechanical protection to the surface of the plants (e.g., hairs, trichomes, thorns, spines, and thicker leaves) or by the production of toxic chemicals such as terpenoids, alkaloids, anthocyanins, phenols, and quinones that either kill or impair the development of the herbivores^14^. Plant secondary metabolites play a key role in resistance against many chewing herbivores, but their role against aphids is less clear. Although aphids may occasionally come into contact with secondary metabolites, their efficacy is generally thought to be dependent on ingestion. Defensive metabolites are produced inside plant cells, and even though defensive compounds can be translocated via the phloem, only some of these compounds are likely to be phloem-mobile in their active form. Since aphids monitor surface features and make short probes to evaluate sample cell contents, this could be a way to incorporate defensive metabolites^15^. To cope with the overabundance of sugars in their diet, aphids have evolved morphological adaptations to exude large quantities of sugars (in honeydew), often together with plant secondary metabolites. Because aphids cause little structural damage to a plant, plant defensive compounds that rely on structural damage for their production are not triggered^16^. Other than the excretion of secondary metabolites together with honeydew, aphid specialists have evolved other traits to deal with specific metabolites, which involve avoidance of uptake by the gut, active elimination from the body cavity, degradation by detoxifying enzymes after uptake, and/or development of insensitive target sites for plant toxins^17–19^. While the mechanisms to deal with defensive alkaloids by aphids is well-studied^16,20,21^, detoxification mechanisms to other defensive compounds are much less explored.

Once on a prospective host, aphids utilise tactile, gustatory cues and probes to ascertain host suitability^22,23^. The epidermis of plant leaves often harbours secondary metabolites and volatiles with a repellent and deterrent activity^24^ and physical structures that influence insect movement and determine plant suitability. Leaf trichomes are among the main defence mechanisms on plant leaves and stems^25,26^. In Solanaceae, we can differentiate two classes according to their function: (1) non-glandular trichomes (types II, III and V), whose fundamental function is to act as physical barriers against water loss by evapotranspiration or impairing the activity of insect pests; and (2) glandular trichomes (types I, IV, VI, VII), which in addition to those same functions have a glandular function producing compounds (volatile or not) that mediate indirect defence against insects^26^. In *Solanum* spp., trichomes are associated with high resistance levels to diverse arthropod species. In particular, the secretion of trichomes IV and VI of several species of tomato are mainly composed of acylsugars, sesquiterpenes, and methyl ketones^27,28^. In addition, glandular trichomes are a source of sugar esters and secondary metabolites like 2-tridecanone that are detrimental to herbivores, and some acylsucroses are also hypothesised to be important deterrents of aphid feeding^26^. Glandular trichomes also release (E)–β-farnesene, the aphid alarm pheromone, promoting 'dispersal behaviour' in aphids^29–33^. Secondary metabolites also contribute to defence against a variety of insects, including whiteflies^34–37^, aphids^38^, spider mites^25^, and leaf miners^39^. Cultivated tomato (in contrast to wild species) lacks many of these secondary metabolites and its glandular trichomes, making them relatively susceptible to a wide range of pests^31,40–42^.

At the forefront of the physiological adaptation of insects to their environment are those enzymatic activities related to energy production and those involved in the biotransformation and detoxification of plant defensive compounds. Both are particularly important for the survival of the insect and its ability to adapt to the changing conditions of the environment^43^. "Omic" technologies and the associated databases are useful for a better understanding of feeding, development, defence pathways, stress factors, and plant defensive compounds in insects^44–47^. However, the information available on molecular biomarkers in phytophagous insects exposed to plant secondary metabolites is limited to date.

**Figure 1:**
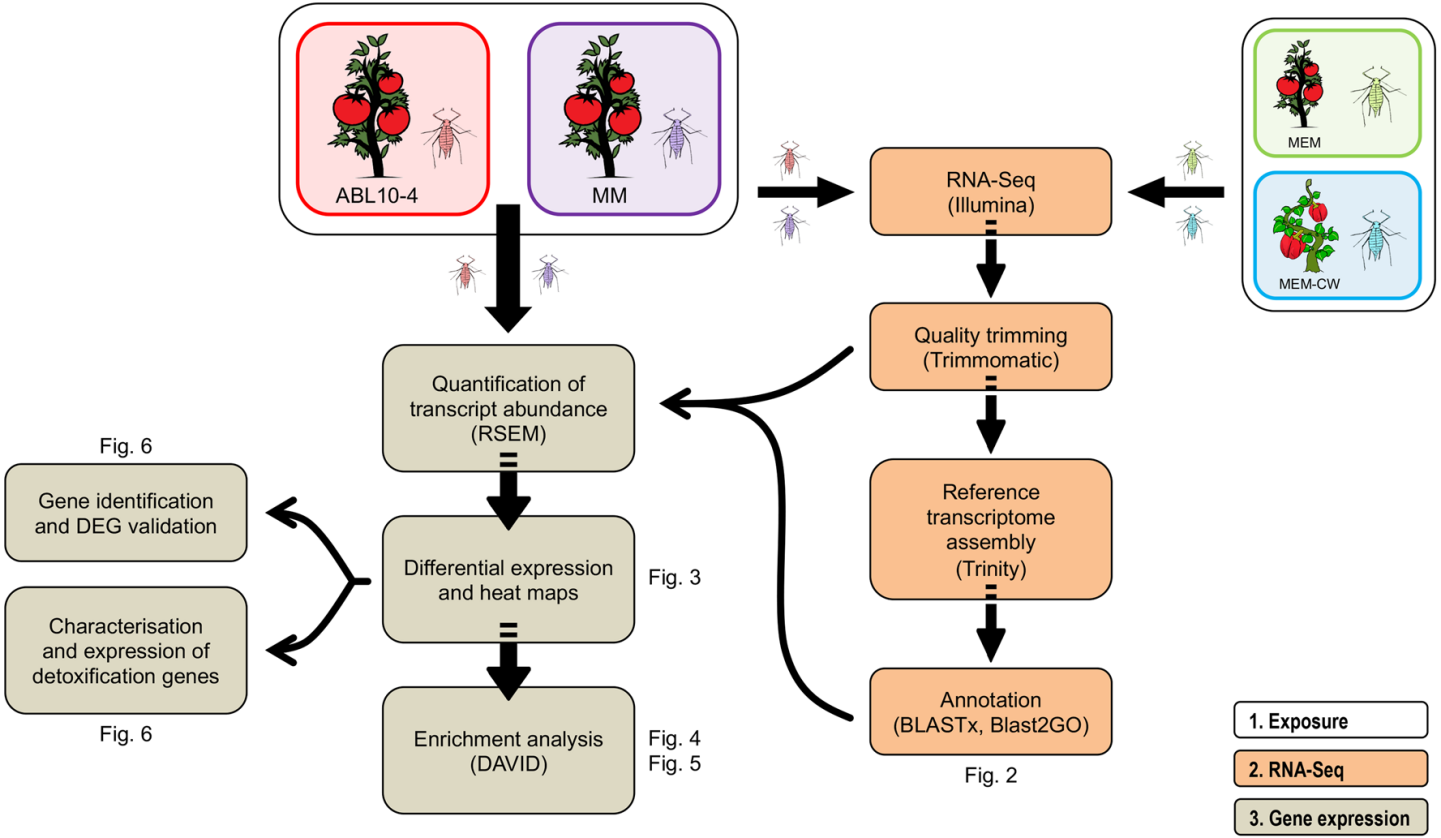
Workflow followed in the present study.

In this study, we used RNA-Seq to compare the molecular response of the aphid *Macrosiphum euphorbiae* when fed ABL10-4 or susceptible Moneymaker, two nearly-isogenic tomato lines with and without resistance traits based on type IV leaf glandular trichomes derived from the wild tomato species *Solanum pimpinellifolium*. For this purpose, we produced a reference transcriptome and quantified transcript abundances of *M. euphorbiae* genes to provide insights into insect gene expression in response to different plant tomato genotypes and trichomes secretions. Furthermore, we aimed to identify a wide range of genes, including those encoding detoxification enzymes induced by plant secondary metabolites and/or insecticides (Fig. 1).

## Methods

### Experimental plants

We used two tomato lines, i.e., 'Moneymaker' (hereafter MM) and its near-isogenic line ABL10-4. ABL10-4 was generated from the initial cross *S. lycopersicum* cv. Moneymaker × *S. pimpinellifolium* acc. TO-937 according Ferrero et al.^41^. The tomato cultivar ‘Moneymaker’ (MM) and its near-isogenic line ABL10-4 were used to treat the aphids. ABL10-4 was generated from the initial cross *S. lycopersicum* cv. Moneymaker x *S. pimpinellifolium* acc. TO-937 followed by five cycles of combined recurrent crosses toward ‘Moneymaker’ and subsequent selfing steps with selection for high type-IV trichome density and acylsugar production, plus two additional final selfing steps. So by using these two genotypes, i.e. the isogenic line ABL10-4 and one parental line MM, we can be sure that differences in aphid responses can be ascribed to the absence or presence of type IV grandular trichomes and acylsacarores and not to any other trait as both materials have the same genetic background (as revealed by QTL analysis and NGS^41^). Moreover, to have a representative sample for the transcriptome analysis, additional aphids clones were reared on bell peppers *Capsicum annum* cv. California Wonder. Seeds were surface sterilised with an aqueous solution of 50% household bleach (30 min), rinsed two times with distilled water and sown on wet filter paper in Petri dishes. After 10 days of germination, seeds were put in seedbeds with autoclaved soil mixture (45% brown and black peat, 45% coconut fibre and 10% pearlite) and tomato and bell pepper plants were grown in a greenhouse (25 °C ± 5 °C). Then, 20 days after germination, plants were transplanted to an 18 cm diameter pot. Plants were watered twice a week with a water-soluble NPK (SO3) [1.98–3, 41–20, (4.46)] fertiliser mixture (Fertiluq) and water-soluble N (Ca-Mg) [4.32 (6.51–4.02)] fertiliser mixture (Fertiluq) with micronutrients were applied. Seeds were obtained from the germplasm bank at the Instituto de Hortofruticultura Subtropical y Mediterranea ‘La Mayora’ (IHSM-UMA-CSIC).

### Aphids colonies

Four aphid populations were used for the reference transcriptome: three colonies reared on tomato (two on susceptible tomato “Moneymaker” -named as MM and MEM, respectively- and one on resistant ABL10-4) and a colony maintained on susceptible pepper (MEM-CW) to maximize the genome coverage of the *M. euphorbiae* expressed genes (Table 1). A total of 12 libraries for three biological replicates (25 insects each) for each population were prepared. Plants received 3 adult females, apterous and viviparous, that came from a single aphid clone. We replicated this set-up 15 times per tomato genotype. The population build-up was monitored every day, and we collected 25 adult individuals for transcriptome analysis after 5 weeks on the plants.

**Table 1:** Aphids samples used for *M. euphorbiae *de novo transcriptome.

Population group	Host	Sample	Read count	GC (%)
MM	Tomato cultivar "Moneymaker"	MM-P1	53,175,708	43.56
		MM-P2	53,291,090	44.44
		MM-P3	58,748,458	43.61
ABL10-4	Tomato isogenic line ABL10-4	ABL10-4 -P1	21,725,650	40.27
		ABL10-4 -P2	44,579,956	47.08
		ABL10-4 -P3	37,381,678	42.93
MEM	Tomato cultivar "Moneymaker"	MEM1	42,960,032	48.62
		MEM2	39,617,224	44.27
		MEM3	40,539,508	45.25
MEM-CW	Pepper cultivar	MEM-CW1	45,487,942	44.23
		MEM-CW2	48,825,974	44.57
		MEM-CW3	52,505,780	43.65

Type of population and read counts per sample after trimming.

For gene expression analysis, samples of aphids reared on MM and ABL10-4 tomato plants were used, with three biological replicates per population (25 adult female each; N = 75 insects per condition).

### RNA extraction for library preparation, sequencing, de novo assembly and annotation

For each sample, RNA was extracted from 25 apterous and viviparous females using TRIzol Reagent (Invitrogen, USA) following the manufacturer's instructions as described in previously^42^.

cDNA libraries were constructed by Macrogen following Tru-Seq Stranded mRNA (Illumina) protocol and sequenced on Illumina Hi-Seq 4000 using a 100 cycles paired-ended protocol. Briefly, poly-T oligo-attached magnetic beads were used to isolate poly(A) + mRNA, using two rounds of purification. The first-strand cDNA was obtained reverse transcribing the cleaved RNA fragments using short fragments of random hexamer primers as templates. The second-strand cDNA was synthesised by removing the RNA template and synthesising a replacement strand, incorporating dUTP instead of dTTP to generate double-strand cDNA. AMPure XP beads were used to separate the double-strand cDNA from the second strand reaction mix. At the end of this process, blunt-ended cDNA was constructed. End repair of the overhangs resulting from fragmentation into blunt ends was performed using an End Repair (ERP) mix. 'A' nucleotide was added to the 3' ends of the blunt fragments to prevent them from ligating to one another during the adapter ligation reaction. Ligation of multiple indexing adapters to the ends of the double-strand cDNA was carried out to prepare them for hybridisation onto a flow cell. Subsequently, PCR-amplification was used to selectively enrich those DNA fragments that have adapter molecules on both ends and create the final cDNA library template.

The raw reads (100pb) were assessed for quality using FastQC^48^. Further, low-quality (Phred value < 33) and adaptor sequences from the raw data were removed using the Trimmomatic (v0.32) tool. Assembly and annotation were performed as described previously^42^.

Finally, BUSCO^49^ was used to perform a quantitative assessment of completeness in terms of expected gene content of the transcriptome using metazoa_odb10.

### Differential expression analysis

Quantification of transcripts abundance from RNA-Seq data was done with the RSEM tool (v1.2.15), mapping the reads to the reference transcriptome created. To reduce systematic bias and avoid statistically erroneous conclusions, we filtered, transformed and normalised the data. Firstly, we filtered the contigs, taking into account all 12 libraries included in the reference transcriptome; contigs for which more than one FPKM (fragments per kilobase transcript length per million mapped reads) value was 0 were excluded from the analyses. Then we proceeded with the comparison of ABL10-4 *vs* MM samples. We transformed the data using Log2(FPKM + 1) and then performed quantile normalisation with the preprocess Core R library (normalise quantiles method^50^). T-tests and calculation of fold change values (FC) were performed to detect gene expression differences among studied conditions (MM and ABL10-4). We considered that a contig was significantly differentially expressed when FC ≥ 2 and *P*-value < 0.05. Clustering analysis through hierarchical clustering (Euclidean Distance, Complete Linkage) and a Heat map was performed by Macrogen using the statistically significant data. Finally, an enrichment analysis was conducted in DAVID^51^, including those contigs that were differentially expressed and successfully annotated, using the reference transcriptome as a background.

### Gene characterisation

The nucleotide sequences for relevant genes involved in detoxification pathways and the oxidative stress response (*cyp4g15*, *cyp6a13-like, cyp6k1-like, cyp4c1*, Cu–Zn *superoxide dismutase (SOD),* glutathione S transferase *(GST)* and *glutathione peroxidase (GPx)*) were obtained from our *M. euphorbiae* reference transcriptome. SnapGene software (www.snapgene.com), BLAST protein tool and DOG V.2 software were used to identify and characterise genes.

### Validation of RNA-Seq results by RT-qPCR

Four sequences from the Differential Expression Genes analysis (DEGs) of *M. euphorbiae* that were differentially expressed in response to the two tomato plants were selected to confirm the variation in their expressions. The same total RNA samples kept on MM and ABL10-4 isolated for RNAseq were used for technique validation. A total of 7 µg of RNA was reverse-transcribed in a 20 µl reaction system in a C1000 Thermal Cycler (Bio-Rad, USA) using iScript Reverse Transcription Supermix for RT-qPCR (Bio-Rad, USA), according to the manufacturer's protocol.

Specific primer pairs for each gene were designed using Primer 3 (version 0.4.0) software^52^ (Supplementary Table 1). Real-time quantitative PCR (qPCR) was performed using a CFX96™ real-time quantitative PCR system (Bio-Rad, USA) with the Quantimix Easy Kit (Biotools B&M Labs, Spain), according to the manufacturer's protocol. Genes encoding actin and the 26S ribosomal subunit were used as endogenous references. Each qPCR was conducted in a 10 μl mixture containing 1 μl of sample cDNA, 0.6 μl of each primer (10 μM), 4,8 μl of nuclease-free water, and 5 μl of 2 × Quantimix Easy Kit. The qPCR cycling parameters were as follows: 95 °C for 30 s, followed by 35 cycles of 95 °C for 5 s and 58 °C for 20 s and 20 s elongation at 65 °C. Melting curve generation was performed from 65 to 95 °C. The mRNA level of each target gene was normalised against the expression of the two reference genes. A variation of the 2^−ΔΔCt^ (Livak) method^53^ was used to analyse relative changes in gene expression with CFX Maestro Software (Bio-Rad, USA). To check reproducibility, the qPCR for each sample was run in duplicate wells, and three technical replicates and three independent biological replicates were performed for each experimental condition.

Statistical analyses were carried out using RStudio^54^. Normality and homoscedasticity of data were assessed with the Shapiro–Wilk's test and the Levene's test, respectively. Since data were not homogeneous or normally distributed, Mann–Whitney U test was used (*p* < 0.05).

### Ethics declarations

This study complies with local and national guidelines.

## Results

### De novo* M. euphorbiae* reference transcriptome

An extensive transcriptome of *M. euphorbiae* was obtained from apterous and viviparous adult females of four different populations adapted to different host plants (Table 1). After trimming, a total of 698.5 million reads were used to construct the reference transcriptome of *M. euphorbiae* and reads per sample ranged from 21 to 58 million. All reads were deposited in the European Nucleotide Archive (ENA) project number PRJEB35133. These reads were assembled into 240,067 transcripts and 189,229 unigenes, with N50 lengths of 771 and 635 nucleotides, respectively (Supplementary Table 2) and with 29,128 contigs longer than 1000 bp. BUSCO^49^ showed 96.8% completeness and high duplication (37.4% complete and single, 59.4% complete and duplicated). Fragmentation was found in 2.2% of genes and 1% were missing.

**Figure 2:**
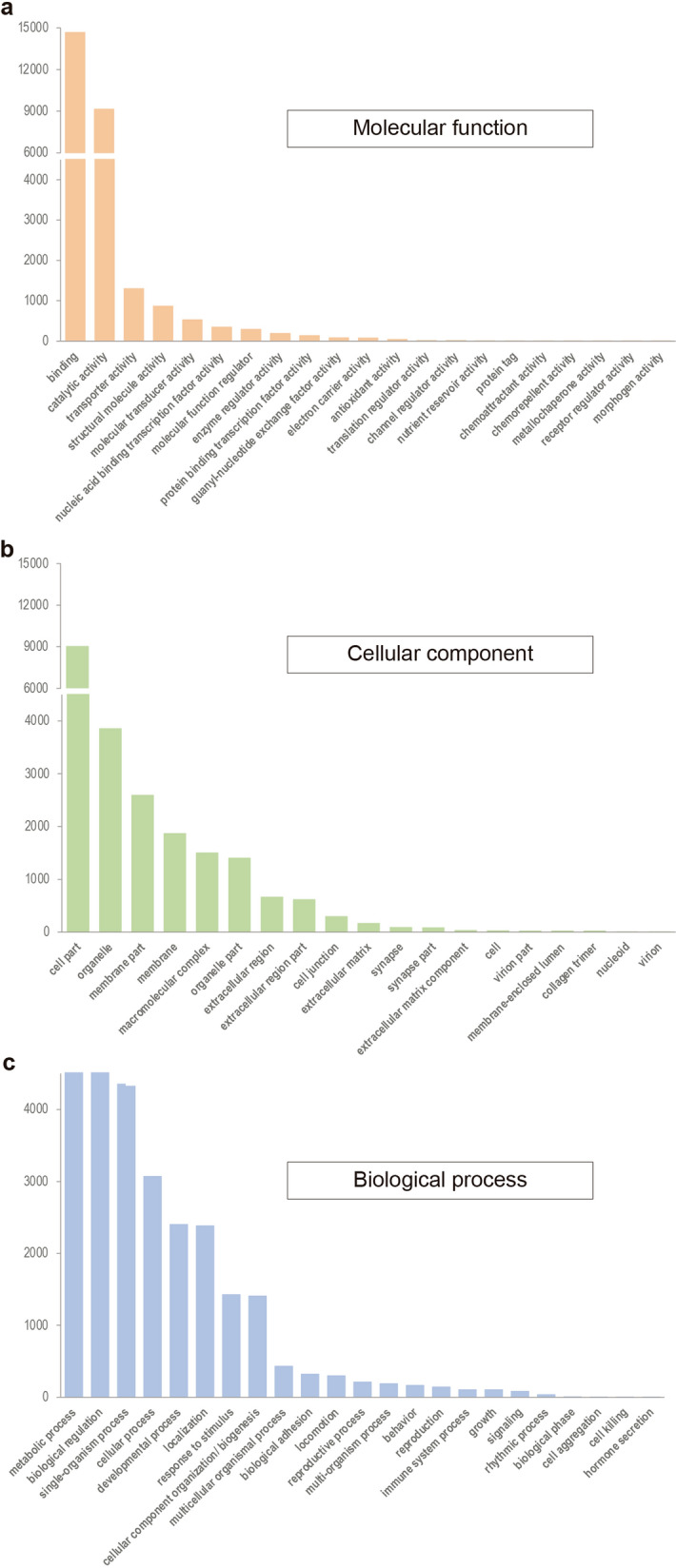
Gene Ontology (GO) classification of transcripts of *M. euphorbiae*. Bar chart describes the distribution of *M. euphorbiae* transcripts into GO categories. Transcripts were annotated in three domains: (**a**) molecular function, (**b**) cellular component, and (**c**) biological process. The y-axis indicates the number of sequences in a given category.

The number of contigs successfully annotated was 87,781 out of 240,067, although the number of unique hits was 32,601. In the categorisation by Gene Ontology (GO), 63 GO Terms were identified within three categories: molecular function (21), cellular component (19), and biological process (23). In addition, GO categorisation shows the predominance of metabolic processes and biological regulation, cell parts (including organelle and membrane), binding and catalytic activity (Fig. 2).

### Differential gene expression analyses

**Figure 3:**
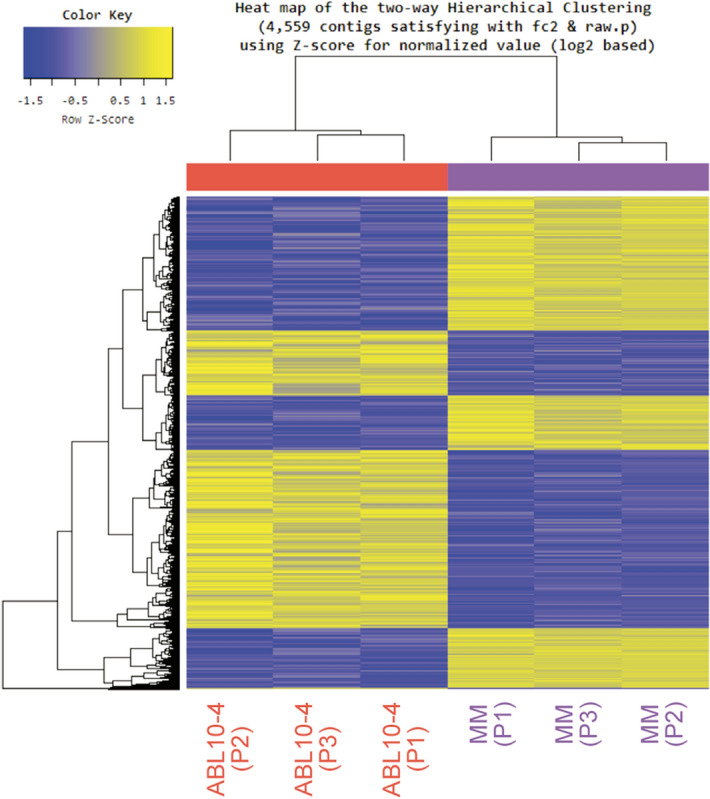
Hierarchical clustering graph of DEGs found between ABL10-4–MM samples. Heat map (performed by Macrogen, with in-house scripts) shows the significant differentially expressed *Macrosiphum euphorbiae* genes when are exposed to Moneymaker and ABL10-4 tomato plants. Each row represents a gene and column represents different aphid samples (3 samples per condition).

To explore the potential effect of natural defences in *M. euphorbiae*, we used data from six DNA libraries of MM (N = 3) and ABL10-4 (N = 3) samples to perform an analysis of the differentially expressed genes (DEGs) (Fig. 3).

**Figure 4:**
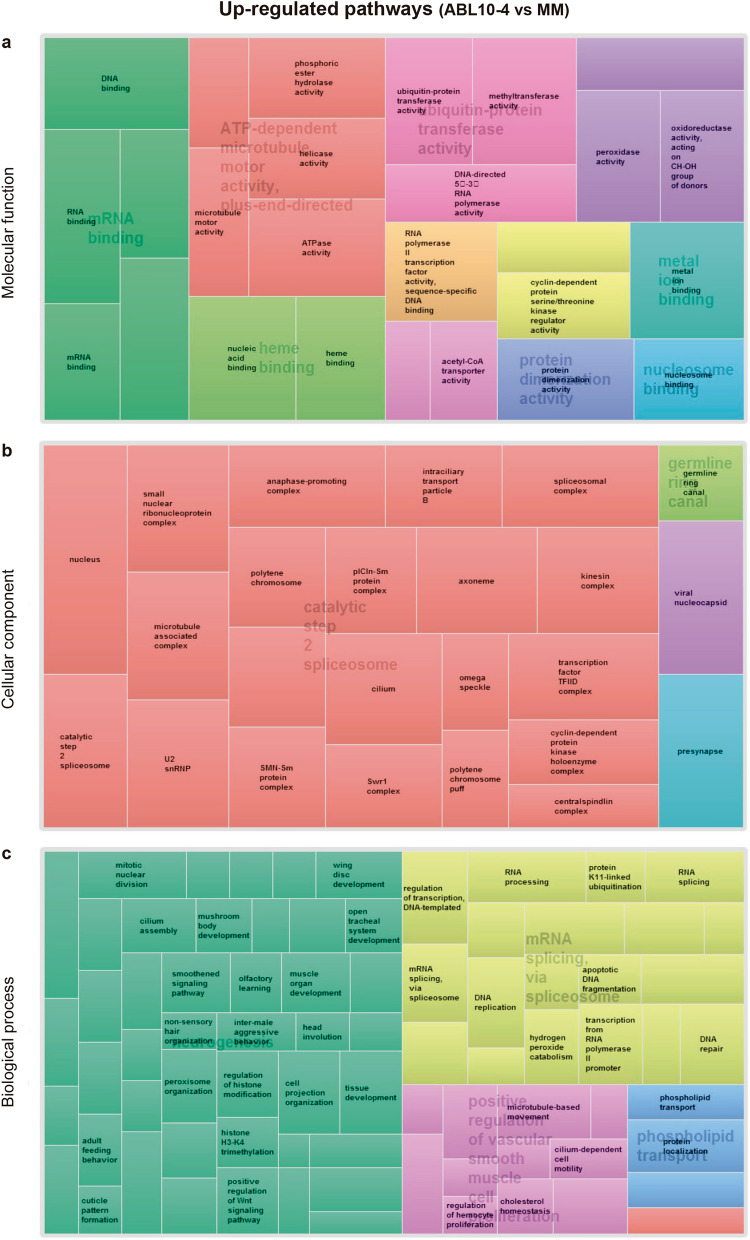
Up-regulated pathways in aphids reared on ABL10-4 comparing to MM. Illustration of superclusters of overrepresented GO-terms visualised in semantic similarity-based treemap views from REVIGO program, for (**a**) molecular functions (**b**) cellular component and (**c**) biological process. Rectangles in the treemaps are size-adjusted to reflect the corrected *P-value* (i.e. larger rectangles represent the most significant GO-terms). Each rectangle in the treemap view has a single cluster for representation. These representatives are further joined together to build superclusters that are related terms and displayed in different colours.

**Figure 5:**
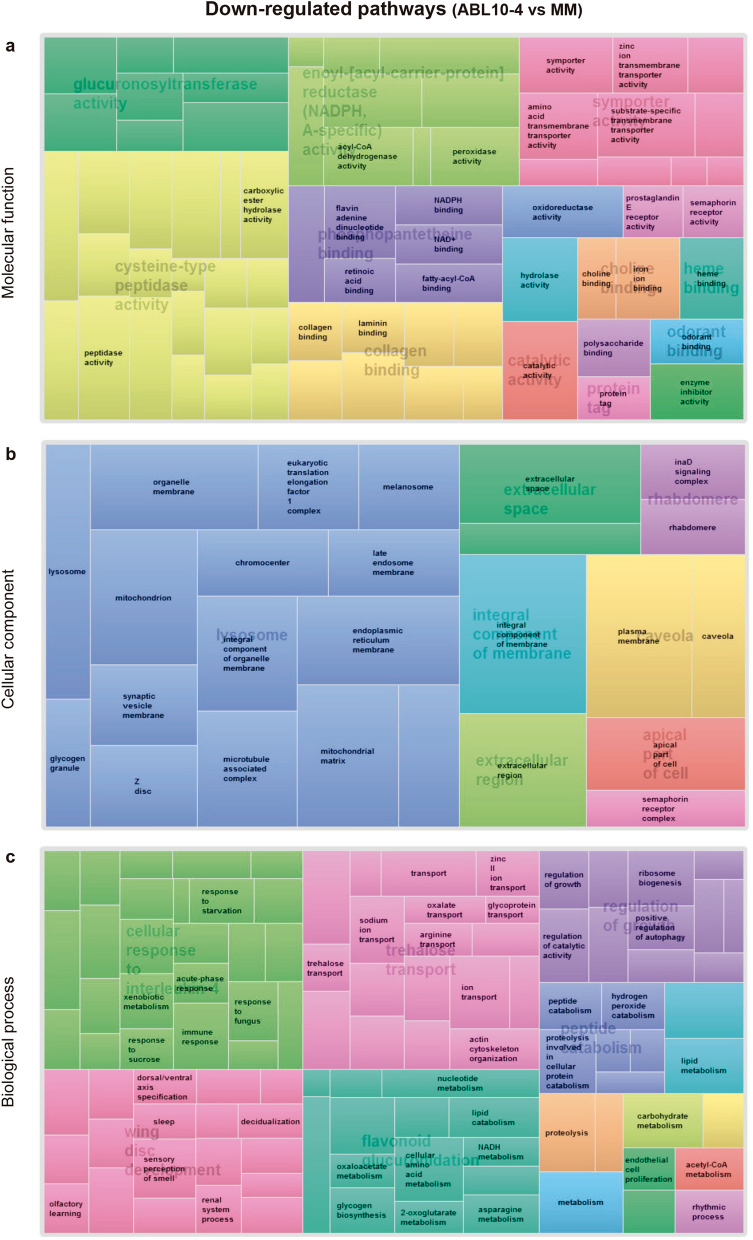
Down-regulated pathways in aphids reared on ABL10-4 comparing to MM. Illustration of superclusters of overrepresented GO-terms visualised in semantic similarity-based treemap views from REVIGO program, for (**a**) molecular functions (**b**) cellular component and (**c**) biological process. Rectangles in the treemaps are size-adjusted to reflect the corrected *P-value* (i.e. larger rectangles represent the most significant GO-terms). Each rectangle in the treemap view has a single cluster for representation. These representatives are further joined together to build superclusters that are related terms and displayed in different colours.

Out of the 240,067 contigs, 23,181 passed the filter following our criteria for FPKM 0 values. Those were, therefore, the basis for DEG analyses. In total, 4,559 contigs were differentially expressed following our criteria FC ≥ 2 and *P*-value < 0.05) in the aphids reared on ABL10-4 (with type IV glandular trichomes) compared with aphids on MM (without type IV glandular trichomes), as revealed in the heat map for the different gene expression profile of those significant contigs (Fig. 3). Out of the 4559 contigs, 2711 showed a BLAST hit, but only 1,817 were unique. Paired comparisons between ABL10-4 and MM highlighted the up-regulation of 658 unigenes and down-regulation of 1159 unigenes (Supplementary Table 3). To illustrate functional differences on aphids reared on MM and ABL10-4 plants, GO enrichment analysis of gene clusters of DEGs were characterised to explore relevant biological functions^55^. The significantly enriched GO terms (*P*-value < 0.05; FDR 5%) were identified both globally and separately for up-regulated and down-regulated genes in aphids reared in ABL10-4 comparing to those reared in MM (Supplementary Figs. 1 and 2, respectively; Supplementary Table 4). Considering the hierarchy structures of GO systems, we used the ReviGO tool to collectively visualise the enriched GO terms for each DEG cluster (Figs. 4 and 5)^56^.

Among the 1159 genes that showed repression, the most enriched categories were those involved in the immune system, the xenobiotic metabolism and oxidative stress responses (e.g., *Cytochromes P450 family, peroxidase*), glycogen and fatty acid biosynthesis (e.g., *Glycogenin*, *desaturase 1*), carbohydrate and lipid metabolism (e.g., *Fatty acid synthase 1*, *phospholipase A2*), regulation of growth and rhythmic process (*Clock, vrille*) (Supplementary Table 3). In addition, genes involved in transmembrane transport also showed a negative regulation on aphids reared on the ABL10-4 genotype (e.g. *ABC transporter B, D and G family members* and UDP glycosyltransferase *Dorothy,* among others).

Among the induced genes in DEG, it is worth noting those related to epidermal formation, such as larval cuticle protein and ecdysteroid biosynthesis (*shade* and *disembodied*, among others). Genes related to adult feeding behaviour, DNA repair and wing development were also up-regulated in ABL10-4 aphids (Supplementary Table 3).

### Quantitative real-time PCR validation of DEG

To validate RNA-Seq measures of expression, RT-qPCR was conducted to compare the expression level of four P450 cytochromes DEGs (three genes down-regulated and one up-regulated) in MM and ABL10-4 aphids (Supplementary Table 5). All of them had unambiguous annotations (Supplementary Table 3). The expression profile of genes selected showed the same results with both approaches, which supports RNA-Seq-based counts of the expression levels obtained by RT-qPCR (Supplementary Fig. 3).

### Characterisation and expression analysis of gene sequences encoding detoxification and biotransformation proteins

We identified genes related to stress response and detoxification pathways i.e., *cyp4g15, cyp6a13-like, cyp6k1-like, cyp4c1, Cu–Zn SOD-like, GST* and *GPx*. A systematic search in the de novo transcriptome of *M. euphorbiae* rendered sequences with open reading frames (ORFs) for these proteins. Two sequences with the complete ORF (*cyp4g15* and Cu–Zn *SOD-like*) and five with incomplete ORFs (*cyp6a13-like, cyp6k1-like, cyp4c1, GST* and *GPx*) were obtained. Sequences were registered in NCBI database as: MT105339 (*cyp4g15*), MT105340 (*cyp6a13-like*), MT105341 (*cyp6k1-like*), MT105342 (*cyp4c1*), MT105343 (Cu–Zn *SOD-like*), MT105344 (*GST*) and MT105345 (*GPx*) (Supplementary Table 6).

**Figure 6:**
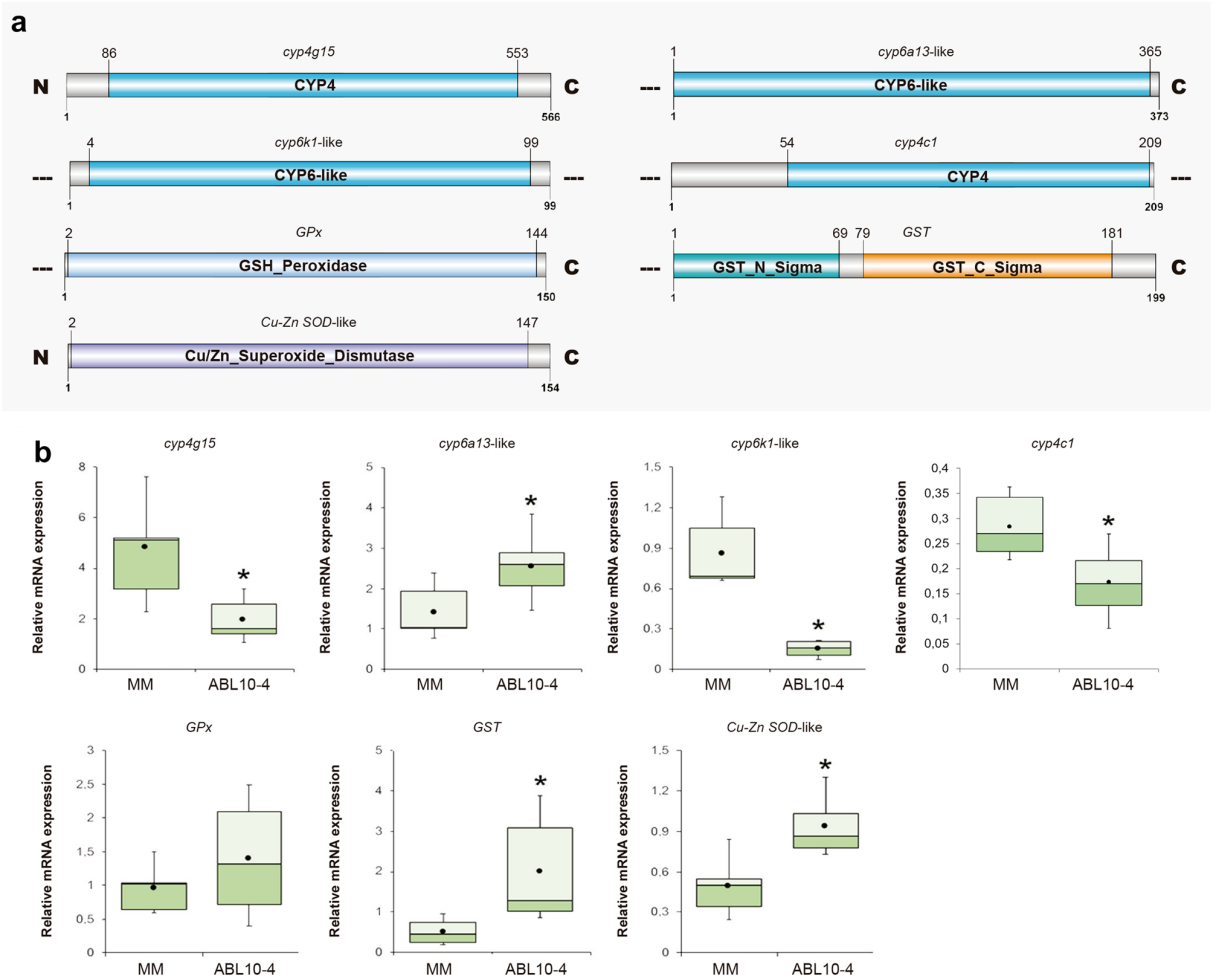
(**a**) Detoxification and oxidative stress proteins identified from de novo transcriptome of *M. euphorbiae*. GenBank Accession numbers are indicated in Supplementary Table 6. Diagram of the protein of *M. euphorbiae* identified as putative mRNAs and their conserved domains. Diagram designed with DOG V.2 software. (**b**) Transcriptional activity of *Cyp4g15*, *Cyp6a13-like*, *Cyp6k1-like*, *Cyp4c1*, *GPx*, *GST*, *Cu–Zn SOD-like* in *M. euphorbiae*. Box and whisker plots represent the expression patterns of *Cyp4g15, Cyp6a13-like, Cyp6k1-like, Cyp4c1, GPx, GST, Cu–Zn SOD-like* measured by real-time RT-qPCR. Box and whiskers represent the 25–75 percentile and the minimum/maximum measured values; mean is represented by a dot; horizontal line separating the lower (dark) and the upper (light) area represents the median. (*) Asterisks indicate significant differences between MM and ABL10-4 aphids, *P* < 0.05.

The relevant domains of each ORF (Fig. 6a) confirm the identity of each de novo characterised protein in *M. euphorbiae*. Supplementary Table 6 showed the percentage of identity of each protein to closest species on databases in BLAST tool (NCBI).

Four of the genes were identified and characterized due to the presence of a Cyp450 domain and some of the most conserved motifs for CYP proteins: heme-binding region (FXXGXRXCXG), meander motif (PXXFXPXXF), K-helix region (EXXR) and C-helix region (WXXXR) (Fig. 6a; Supplementary Table 5). The ORF was complete in one of these sequences (cyp4g15), while the other three were incomplete. A DNA of 1,701 bp coded the complete ORF; it had a length of 566 aa and it shared 99% and 98% identity with CYP4g15 of *Acyrthosiphon pisum* and *Myzus persicae*, respectively. This amino acid sequence contained the C-helix region conserved motif (WRAHR; residues 141–145), the K-helix sequence motif (ETLR, residues 421–424), the meander motif region (PNPEVFNPDNF; residues 472–482) and, the heme-binding sequence motif (FSAGPRSCVG; residues 498–507).

The first incomplete sequence was a 1,122 bp DNA with an ORF of 373 aa that contained the heme-binding domain (FGDGPRHCIG), the K-helix motif (ETHR) and the meander motif region (PETFDPERF). It shared 97% and 95% identity to CYP6a13 from *Acyrthosiphon pisum* and *Myzus persicae*, respectively. The second Cyp450 was 299 bp in length, and the protein had 99 aa and contained the K-helix motif (ETER) and the meander motif region (PLRFDPERF). The highest identity (91%) was with the CYP6k1 of *Acyrthosiphon pisum*. The last cytochrome identified belonged to the Cyp4 family, and the size of the DNA was 630 bp. It coded for a 209 aa protein and contained the C-helix motif (WQTR) and the meander motif region (PLRFDPERF). It showed 98% and 92% of identity with CYP4c1 from *Acyrthosiphon pisum* and *Myzus persicae*, respectively.

Different proteins were identified concerning biotransformation processes. First, the complete ORF (465 bp) of a superoxide dismutase was identified coding a protein of 154 aa. This protein contained the two Cu/Zn-SOD family signature sequences: GMHIHQFGDNT (residues 45–55) and GNAGARPACGVI (residues 139–150) and shared 63% identity with Cu–Zn SOD of *Macconellicocus hirsutus*. Next, the incomplete ORF of GST covered a region of 199 aa; it was 629 bp in length, and it shared 76% identity to GST Sigma-like from *Aedes aegypti*. The GSH binding site (G-site) on the domain GST_N_Sigma (residues 1–69) and the substrate binding pocket (H-site) on the domain GST_C_Sigma (residues 79–181) were found in this amino acid sequence. Both regions contained all the residues that compose these conserved features.

Finally, the last incomplete sequence was a 454 bp DNA coding an ORF of 150 aa. It shared 53% identity to glutathione peroxidase from *Melanaphis sacchari* and contained the three highly conserved motifs of GPx: GKVVLVVNTASKCG (GPx signature 1), ILAFPCNQF (GPx signature 2) and WNFEKF (conserved active site motif).

Analyses of the transcriptional activity of identified targets were carried out in viviparous females exposed to MM and ABL10-4 plants, respectively. In terms of detoxification processes, different changes in expression profile were observed depending on the gene. Compared to MM aphids, significant repression of *cyp4g15*, *cyp6k1-like* and *cyp4c1* in ABL10-4 (up to 5.6-fold, 2.7-fold and 1, ninefold respectively) were observed in individuals reared on ABL10-4 (*P* = 0.001, *P* = 0.011 and *P* = 0.019, respectively). In contrast, a significant induction of *cyp6a13-like* expression (a mean of 5.7-fold; *P* = 0.008) was detected in these aphids (Fig. 6b).

Gene expression of *GPx*, *GST* and *Cu–Zn SOD-like*, involved in biotransformation processes, showed a similar tendency to that observed for *cyp6a13-like*. The overexpression of *GST* and *Cu–Zn SOD-like* were significant (*P* = 0.015 and *P* = 0.017, respectively), while *GPx* *P*-value was in the limit of significance (*P* = 0.0503) in aphids after exposure to the ABL10-4 plants in comparison to the MM condition (Fig. 6b).

## Discussion

Comparative analysis of genomic information enables identifying novel target genes. This traditionally occurs through genomic, transcriptomic, and metabolomic analyses of model organisms; however, there is little or no genomic information available for many agricultural pests. In this study, we first built a de novo transcriptome from females from four populations of *M. euphorbiae* (Table 1) as the first resource for downstream applications, including detecting potential biomarkers related to the response to plant defences. Then, using two tomato lines that only differ in the presence of type IV glandular trichomes (and exacerbated production of acylsugars), we could infer the effect of plant defences and their associated exudates on the aphids, shedding light on the mechanisms of interaction between the plant and the insect.

The number of genes successfully annotated (32,601) in our de novo *M. euphorbiae* reference transcriptome was in line with the total number of genes found in other studies with aphids with whole transcriptome sequences available, such as *M. euphorbiae* with 20,254 annotated contigs^57^, *Myzus persicae* with 33,543 annotated transcripts^58^, *Sitobion avenae* salivary glands with 10,776 annotated unigenes^59^, 27,112 annotated transcripts in the de novo transcriptome of the mustard aphid *Lipaphis erysimi*^60^ and *P. solenopsis* with 38,725^61^.

To explore the potential effect of natural defences through the exposure to glandular trichomes, enrichment and DEG analyses comparing aphids reared on MM and ABL10-4 were performed, leading to 658 up-regulated unigenes and 1,159 down-regulated unigenes in aphids reared on ABL10-4. The most abundant GO Terms for Biological Processes among the up-regulated and down-regulated transcripts (Supplementary Figs. 1 and 2, respectively) give the major biological processes attributed to plant defences.

### Comparison of enriched pathways in aphids reared on ABL10-4 and MM

"Oxidation–reduction" was one of the most abundant biological processes identified among the down-regulated transcripts in ABL10-4 aphids compared to MM aphids. Transcripts involved in oxidation–reduction were related to proteins involved in a diverse range of pathways: metabolism of insect hormones (CYP18a1 and CYP305a1); breakdown of synthetic insecticides *(*CYP6a14*)*; growth and development (genes coding inosine-5'-monophosphate dehydrogenase, glucose dehydrogenase and chorion peroxidase, respectively), response to oxidative stress (sulfiredoxin gene-*srx*), and immune response (peroxidasin (*pxn), prophenoloxidase 2 (PPO2)*) (Table 2).

**Table 2:**
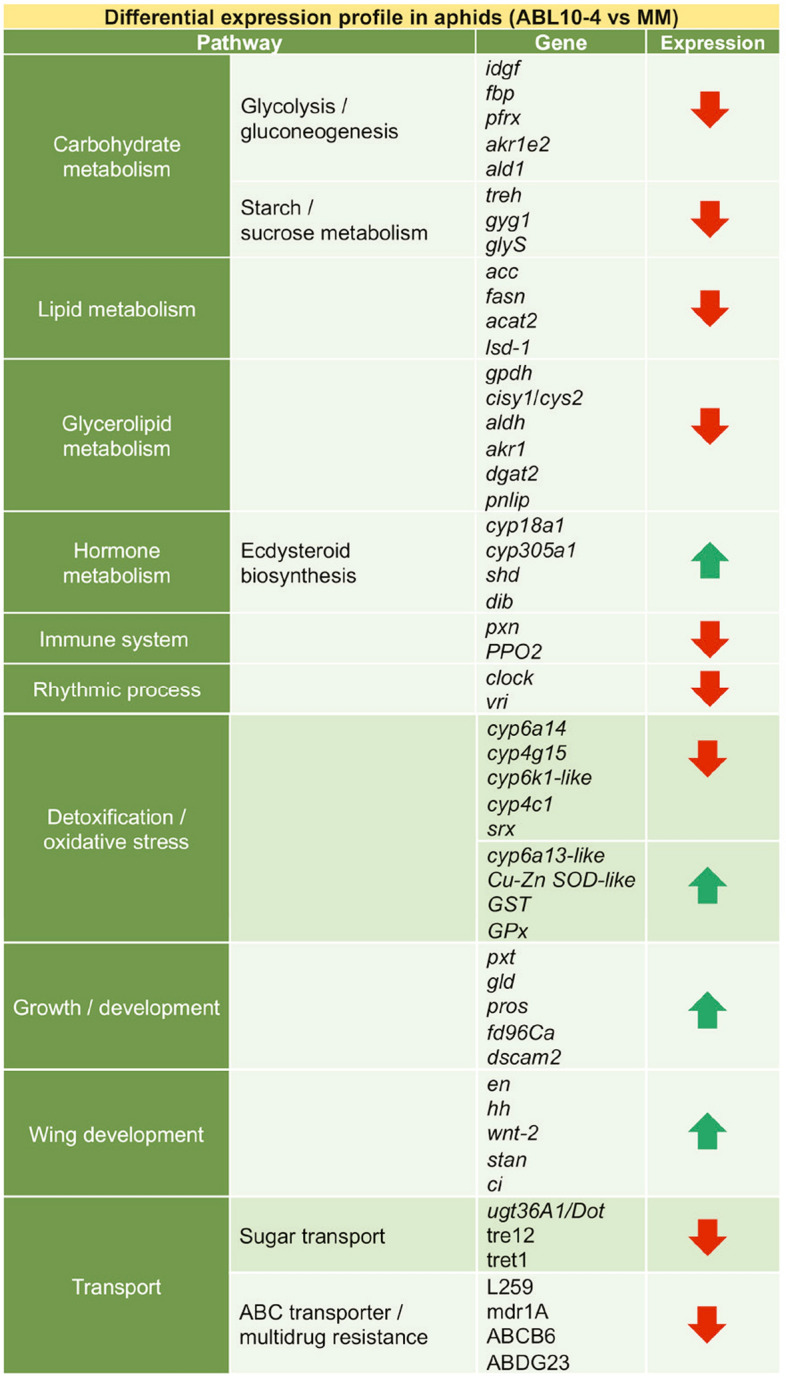
Main biological functions associated with relevant differentially expressed (DE) genes.

Summary of results of differential expression profile in aphids reared ABL10-4 and MM tomato plants. Table shows relevant DE genes from enriched pathways in aphids reared on ABL10-4 compared to those reared on MM, as described in “Results” and “Discussion” sections.

### Transport

Genes involved in transport also showed a negative regulation on aphids reared on the ABL10-4 genotype. 60 down-regulated transcripts were identified related to oxalate, trehalose, glycoproteins and ions transport. Moreover, 37 genes involved in transmembrane transport, including insecticides (e.g., ABC transporter B, D and G family members and multidrug resistance-associated proteins), and sugar transmembrane transport (UDP glycosyltransferase Dorothy and other glucuronosyltransferase genes) were affected (Table 2).

### Carbohydrate and lipid metabolism

"Carbohydrate metabolism" was also an enriched down-regulated pathway in ABL10-4 with respect to MM. Similarly, transcripts associated with chitinase (*idgf*), fructose-1,6-bisphosphatase (*fbp)*, fructose-2,6-bisphosphatase (*pfrx*), 1,5-anhydro-D-fructose reductase (*akr1e2*), and fructose-bisphosphate aldolase (*ald1*) gene expression were also down-regulated in ABL10-4 compared to MM, among other interesting genes (Table 2; Supplementary Table 3). These transcripts, which code for enzymes related to glycolysis and gluconeogenesis, are expected to be induced in feeding aphids^60,62^. Other transcripts related to starch and sucrose metabolism -trehalase (*treh*), glycogenin-1 (*gyg1*), and glycogen synthase (*glyS*)- were also repressed in ABL10-4 aphids. A significant down-regulation of chitinase and fructose, 1,6-bisphosphatase genes has been described in *Liphapis erysimi* under starvation^60^. Our analyses indicate that glycogen storage, gluconeogenesis and glycolytic processes took place normally in MM aphids, while in ABL10-4 were affected, underlying an inhibition or impairment in feeding due to the presence of type IV trichomes and their secretions.

In the same direction, the observed shifts in the expression of genes related to lipid metabolism point at aphid starvation on ABL10-4. In this sense, relevant genes of fatty acids biosynthesis, elongation and degradation pathways -acetyl-CoA carboxylase (*acc*), fatty acid synthase (*fasn*), acetyl-CoA acetyltransferase (*acat2*) genes- were significantly repressed in aphids reared on ABL10-4 compared to MM (Fig. 4; Supplementary Table 3).

In ABL10-4 aphids, we also detected down-regulated genes related to the glycerophospholipid metabolism included *gpdh* (glycerol-3-phosphate dehydrogenase), *cisY1* and *cysY2* (citrate synthase 1 and 2, respectively), together with a decrease in the expression of aldehyde dehydrogenase (*aldh*), aldo–keto reductase *(akr1)*, diacylglycerol o-acyltransferase 2 (*dgat2*) and pancreatic triacylglycerol lipase (*pnlip*), which are involved in the glycerolipid metabolism. Furthermore, *ppap2a* (phosphatidic acid phosphatase 2A), involved in the synthesis of triacylglycerols (TAGs), was also down-regulated in ABL10-4 aphids. In insects, TAGs are stored in specialised lipid droplets, which are relevant for passive storage and also actively participate in fat and energy metabolism^63^. Our results show concomitant repression of lipid storage droplet protein 1 (*LSD-1*) gene transcriptional activity, which underlies how the lipolytic machinery is compromised in the aphid. Lipids are known to play critical roles in energy homeostasis, membrane structure, and signalling. Triglycerides, along with glycogen and protein granules, are the major component of the lipid droplets and occupy most of the intracellular space in the fat body of insects^64^. The level of reserves accumulated in the fat body modulates several essential aspects of the insect's life, such as the rate of insect growth, the timing of metamorphosis, and egg development^65^. The observed results at the transcriptomic level on carbohydrate, energy and lipid metabolism genes show the inhibition of the corresponding metabolic pathways and, consequently, point at the metabolic basis for the impairment observed in feeding, growth, oviposition in aphids reared on ABL10-4 plants^42^.

### Development

Interestingly, the repression observed in genes related to carbohydrate, lipid and energy metabolism is accompanied by the up-regulation of developmental genes. Homeobox protein genes (*prospero (pros)*, *fork head domain-containing protein FD4 (fd96Ca), dscam2*) were significantly induced in ABL10-4 aphids. Wing forms can be an induced adaptive response of aphids to environmental changes, with starvation being a well-known condition for inducing wing development in aphids^60,66–68^. Wingless females feeding on deteriorating plant sources promoted the production of winged offspring in *Aphis craccivora* and *A. pisum*^69–72^. Wing development was an enriched (up-regulated) biological process in aphids exposed to glandular trichomes. Genes involved in wing development, some of them identified by Brisson et al.^73^ in *A. pisum* (i.e., *engrailed (en)*, *hedgehog (hh), wnt-2, protocadherin-like wing polarity protein (stan)* and *cubitus interruptus (ci)*), were also induced in ABL10-4 reared aphids (Fig. 4; Supplementary Table 3).

### Oxidative stress response and detoxification

The effect of type IV glandular trichomes goes beyond changes in the energy and lipids metabolism and developmental changes (induction of winged morphs). However, it also implies changes in the capacity of aphids to deal with oxidative and toxic stress, which underlies the toxic character of the blend of secondary metabolites (mainly acylsucroses but also other metabolites) produced by glandular trichomes and affecting the aphids at a different level.

Response to oxidative stress was also down-regulated on aphids fed ABL10-4. Aphids reared in MM showed higher levels of oxidative stress-related transcripts, while these were compromised in ABL10-4 aphids. Some saliva genes protect aphids against reactive oxygen species (ROS) produced by plants after insect attack. Among them, catalases (CATs) and peroxidases (Pxd) convert H_2_O_2_ into water and oxygen^74^, and glutathione S-transferases (GSTs) belongs to the Phase II biotransformation process and detoxifies secondary oxidation products generated from ROS^75^. Activity levels of these enzymes in insects are believed to be crucial factors in determining their resistance to a broad spectrum of toxic chemicals^76^. Comparisons of activities of genes coding for antioxidant enzymes in aphids reared on ABL10-4 and MM shed some light on how aphids respond to oxidative stress. Genes coding glucose dehydrogenase (*gld*), glucose oxidase (*gox*) and ß-galactosidase (*glb*), associated with aphid saliva^78^, were significantly repressed in ABL10-4 aphids. Trehalases, responsible for the hydrolysis of trehalose to glucose under stress conditions^77^, are also associated with environmental stress, and were downregulated in ABL10-4 aphids (Supplementary Table 3). Peroxidase acts as an antioxidant enzyme, so peroxidase detected in aphids may protect aphids from oxidative stress caused by plant metabolites, detoxifying exposed organisms and playing an essential role in suppressing ROS production and ROS-induced plant defence responses^78,79^. All these changes suggest that the capacity to respond to oxidative stress is compromised in aphids exposed to glandular trichomes. The lower gene transcriptional activities found in ABL10-4 aphids suggest that these responses pathways do not activate, maybe as a consequence of starvation. It has been described that plant genotype had a significant effect on aphid's choice. Data of no-choice assay performed under the same conditions described the preference of aphid choice for MM (70%) comparing to that of aphid choosing an ABL 10–4 plant (30%)^40^. A clear effect of plant genotype on the selection rate was also observed under free-choice conditions, when aphids could choose either MM or ABL 10–4^42^. Since aphids seem not to feed on ABL10-4 plants as regularly as on MM plants, they might not induce the secretion of detoxification proteins located in saliva to response to oxidative stress provoked by plants.

Detoxification systems are one of the different physiological pathways that may be affected by toxicants. To date, information about the molecular effects of natural plants defences and sensitive targets of exposure to these secretions in aphids is scarce. Cytochrome P450 monooxygenases (P450s, CYP3 and CYP4 clade) constitute essential metabolic systems since they are involved in the oxidative detoxification of plant secondary metabolites and synthetic insecticides^80^. Our findings show a differential response of cytochrome P450 genes to the secretions of ABL10-4 plants. The decrease of some cytochromes (*cyp4g15*, *cyp4c1* and *cyp6k1-like*) could be caused by oxidative stress. The up-regulation of the gene coding for cytochrome *cyp6a13-like* could also be involved in the biotransformation response of aphids exposed to the trichomes' secretion of ABL10-4 plants. The enhanced transcriptional activities of genes coding cytochrome P450 monooxygenases conferring insecticide resistance have been well described in insects such as *N. lugens*^81^, *B. tabaci*^82,83^ and *M. persicae*^84^. However, to date, no data about detoxification genes and their role in insect response to plant defences have been reported in *M. euphorbiae*. Recently, it has been described that the suppression of some CYP genes increased the sensitivity of resistant *A. gossypii* to pesticide^85^. In this sense, the repression of the expression of most of the CYP genes analysed in *M. euphorbiae* exposed to ABL10-4 could be related to the higher sensitivity of individuals to this plant tomato genotype and the significant changes observed at the physiological/behaviour level.

Regarding GST, it is an abundant antioxidant involved in the detoxification of many xenobiotics. GST catalyses the transformation of electrophilic compounds to less toxic substances, by conjugating them to GSH^86^. The increased expression of genes encoding glutathione-S-transferase (*GST*), superoxide dismutase (*SOD*), and glutathione peroxidase (*GPx*) might be due to antioxidation defence and detoxification mechanisms. GPx is important in decomposing peroxide and protecting the integrity of the structure and function of the membrane by eliminating the harmful metabolite peroxide and interrupting the chain reaction of lipid peroxidation^87^. SOD helps to facilitate the transformation of superoxide anion radicals to hydrogen peroxide (H_2_O_2_). The increased SOD activity in the *M. euphorbiae* might be a compensation mechanism against secondary metabolite intoxication from trichomes. Moreover, some aphid effectors have been identified having a GST and SOD activities and whilst GSTs have previously been mainly associated with resistance to insecticides, the findings described here underpin the role of GSTs, SOD and GPx in manipulating plant defences when delivered into the plant^88,89^.

## Conclusions

The transcriptome we generated represents a valuable genomic resource for screening potential gene targets in *M. euphorbiae.* Seven novel genes related to detoxification mechanisms and oxidative stress were de novo identified and showed differential expression in *M. euphorbiae*, proving to be effective potential biomarkers at the molecular level to better understand the physiological response of aphids to trichome secretions and plant defences. Furthermore, the observed differences in the transcriptome between the aphids reared in the two tomato lines give insights into the mechanisms responsible for the resistance mediated by glandular trichomes in tomato. Starvation together with the effect of glandular trichomes (and acylsucroses) results in the repression of genes related to carbohydrate and lipid metabolism, genes involved in response to oxidative stress genes, the induction of developmental genes responsible for the appearance of winged morphs, together with important effects in detoxification pathways. Our results demonstrate the detrimental effect of glandular trichomes (type IV) on the aphid and put forward their mode of action. Given the prevalence of glandular trichomes in cultivated Solanaceae*,* and some of the investigated molecular biomarkers in insects in general, our results provide relevant mechanisms to understand the effect of trichomes not only on herbivorous insects but also on other trophic levels.

## Supplementary Information

Supplementary Information 1.

Supplementary Information 2.

Supplementary Information 3.

## Data Availability

The datasets generated and/or analyzed during the current study have been deposited in the European Nucleotide Archive repository (ENA, https://www.ebi.ac.uk/ena/browser/home; project number PRJEB35133). Gene sequences were registered in NCBI database (https://www.ncbi.nlm.nih.gov/) with the following NCBI accession numbers: MT105339, MT105340, MT105341, MT105342, MT105343, MT105344 and MT105345.
