# Supplementary material for: Transcriptome analysis of aphids exposed to glandular trichomes in tomato reveals stress and starvation related responses
Source: Sci Rep. 2022 Nov 23;12:20154. doi: 10.1038/s41598-022-24490-1 (PMC9684535; doi:10.1038/s41598-022-24490-1)
Supplement: Supplementary file 1 — Supplementary Information 1. [file 41598_2022_24490_MOESM1_ESM.docx]

s
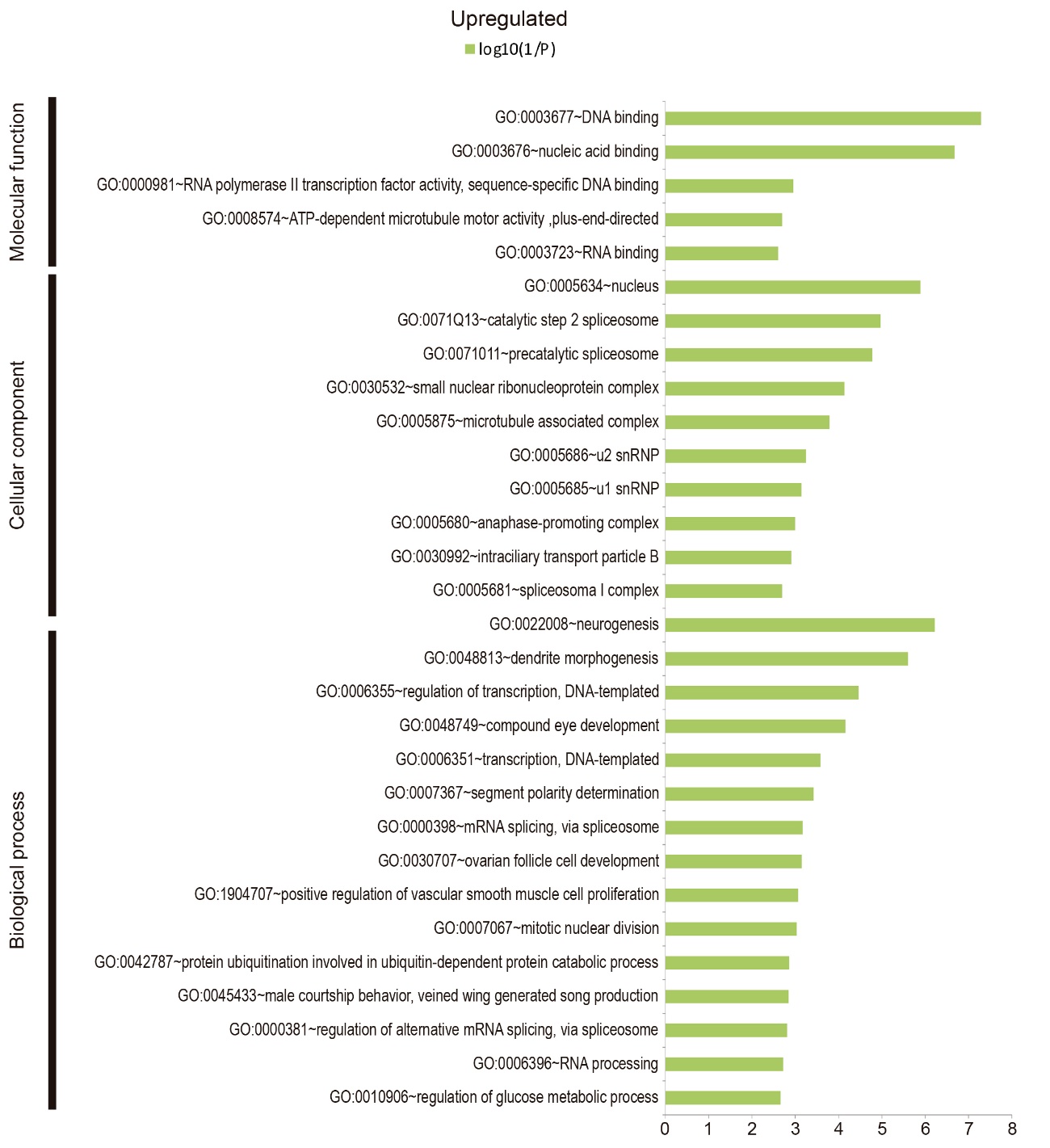


**Supplementary Figure 1. Enriched GO Terms in up-regulated genes (ABL10-4 aphids vs MM aphids).** GO enrichment analysis obtained with differentially up-regulated genes (DEGs). The results were summarized in three major categories, including biological process, cellular component, and molecular function. Graphics represent the significantly enriched (FDR 5%) GO terms obtained from up-regulated DEGs in ABL 10-4 aphids when compared to MM aphids. P values are represented as log10(1/P) to facilitate visualization.

**
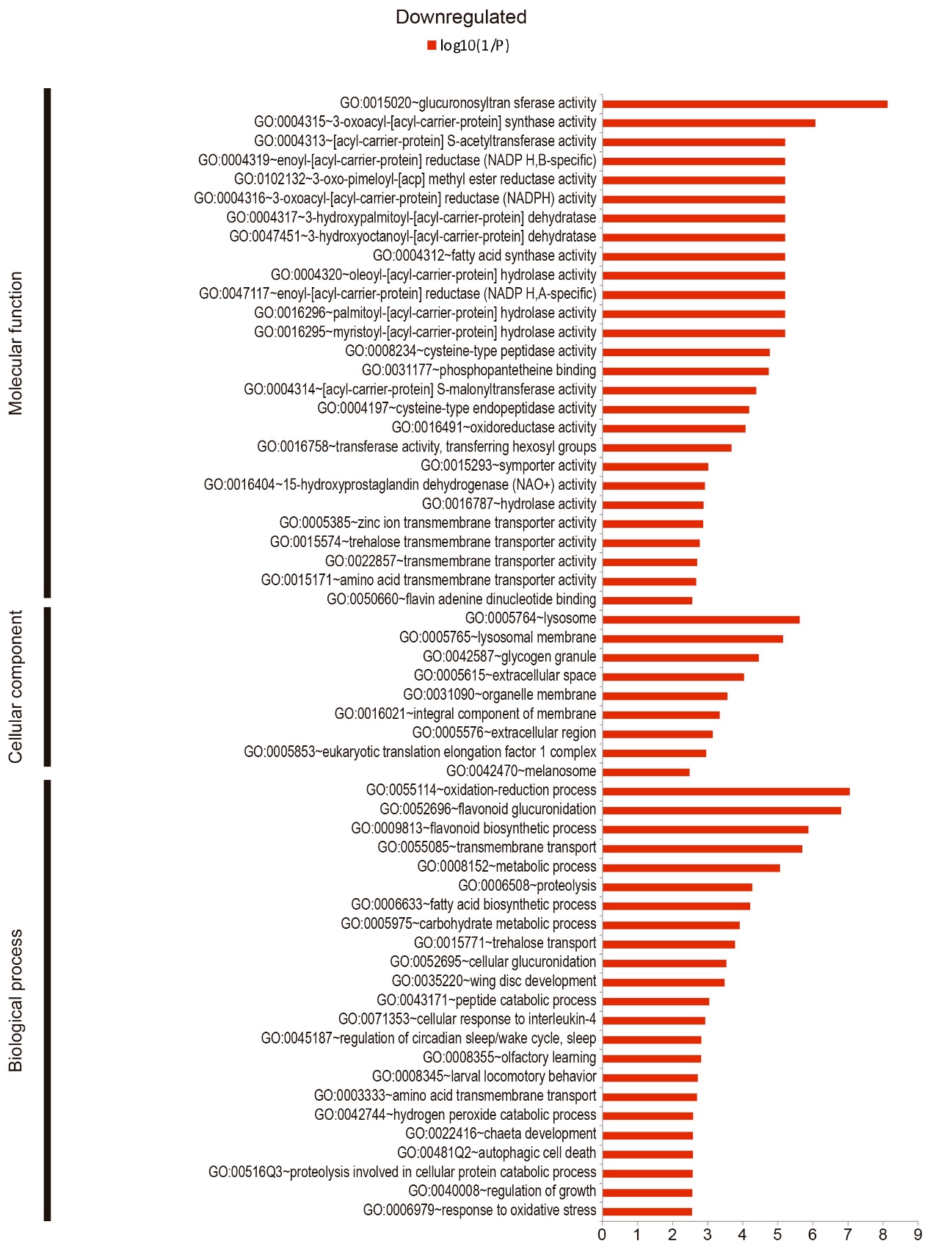
**

**Supplementary Figure 2. Enriched GO Terms in down-regulated genes (ABL10-4 aphids vs MM aphids).** GO enrichment analysis obtained with differentially down-regulated genes (DEGs). The results were summarized in three major categories, including biological process, cellular component, and molecular function. Graphics represent the significantly enriched (FDR 5%) GO terms obtained from down-regulated DEGs, in ABL 10-4 aphids when compared to MM aphids. P values are represented as log10(1/P) to facilitate visualization.


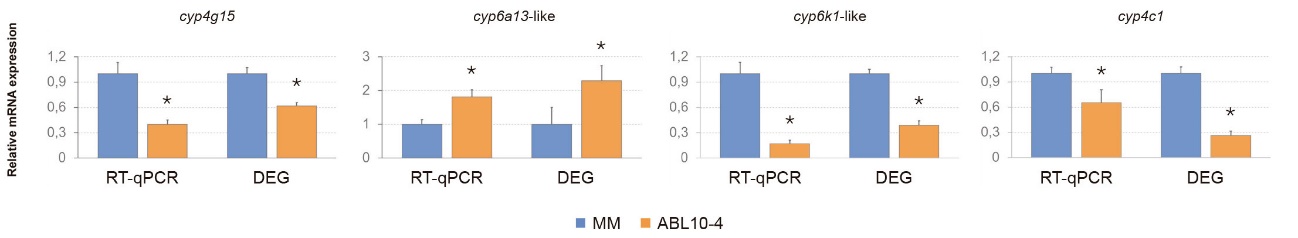


**Supplementary Figure 3.** **Transcriptome data validation by RT-qPCR.** Comparison of *M. euphorbiae* gene expression results between RNA-Seq and RT-qPCR. Error bars indicate SE. Normalization was done using *actin* and *26S* as the reference genes. (*) For each technique, asterisks indicate significant differences between MM and ABL10-4 aphids, P<0,05.

**Supplementary Table 1. Primers used for cDNA sequencing and Real Time qPCR of genes studied in *M. euphorbiae (Me*)*.*** Forward (F) and reverse (R) sequences, length of amplified fragments and origin of primers when corresponds.

| Gene | Primer sequence (5’-3') | Fragment size |
| --- | --- | --- |
| *Cyp4g15* | **F** GGGTGGCATTTTTGGACTTG  **R** CGGCTGCTGTTGTATCGTGA | 120 bp |
| *Cyp6a13-like* | **F** TTCCGAGACTGTAACTGCCACA  **R** GTCCATCGCCAAACGGTAAA | 407 bp |
| *Cyp6k1-like* | **F** AACATACACTTTACCCGACGAATCA  **R** GCCATTAGGTCGTTGAGATTTTTG | 160 bp |
| *Cyp4c1* | **F** TGCCACGAGGATATTTGACTGA  **R** TCGTTTGCCATTTTTGACTTCC | 279 bp |
| *SOD* | **F** CTGATGCACTCCAAAAAGCA  **R** AACTCCAACAGCAGCGACTT | 86 bp  (Park et al., 2012) |
| *GST* | **F** AACGGTCACTTCGCTCTTGG  **R** GGATGGTCGGCTGTCAAATC | 107 bp |
| *GPx* | **F** AAGTGTGGTTACACAGCTAAGCATT  **R** GATATCCAAATTGATTACACGGAAA | 112 bp  (Nair et al., 2012) |
| *Actin* | **F** GATGAAGATCCTCACCGAACG  **R** CGGAAACGTTCATTACCG | 201 bp  (Martínez-Guitarte et al., 2007) |
| *26S* | **F** TTCGCGACCTCAACTCATGT  **R** CCGCATTCAAGCTGGACTTA | 220 bp  (Planelló et al., 2011) |

**Supplementary Table 2.** Summary of *M. euphorbiae* transcriptome assembly.

| Sample | Merge | |
| --- | --- | --- |
|  | All transcript contigs | Only longest isoform per 'gene' |
| Total trinity 'genes' | 189,229 | 189,229 |
| Total trinity transcripts | 240,067 | 189,229 |
| Percent GC | 40.28 | 40.99 |
| N90 | 244 | 235 |
| N80 | 308 | 284 |
| N70 | 405 | 356 |
| N60 | 550 | 464 |
| N50 | 771 | 635 |
| N40 | 1,100 | 912 |
| N30 | 1,593 | 1,357 |
| N20 | 2,247 | 2,059 |
| N10 | 3,227 | 3,105 |
| Maximum contig length | 15,848 | 15,848 |
| Minimum contig length | 201 | 201 |
| Average contig length | 563.74 | 513.03 |
| Total assembled bases | 135,334,529 | 97,079,564 |

**Supplementary Table 3.** Complete dataset of differential expression analysis (DEG) of aphids exposed to ABL10-4 and MM plants.

Attached as supplementary information (.xls file)

**Supplementary Table 4.** Most significantly enriched GO terms in aphids reared on ABL10-4 plants compared to those reared in MM.

Attached as supplementary information (.xls file)

**Supplementary Table 5.** List of differentially expressed CYP transcripts detected by RNA-Seq between aphids exposed to ABL10-4 and MM tomato plants.

| **RNA-seq (ABL10-4 vs MM)** | | | |
| --- | --- | --- | --- |
| **Contig ID** | **Gene identification** | **Foldchange (Fc)** | **P-value** |
| **c234766_g1_i1** | *Cyp4c1* | -9,0422372 | 0,00034929 |
| **c254058_g1_i1** | *Cyp4g15* | -7,3997128 | 0,00405308 |
| **c300320_g1_i3** | *Cyp6a13-like* | 2,90562582 | 0,0301665 |
| **c307953_g1_i2** | *Cyp6k1-like* | -12,456248 | 0,00014581 |

**Supplementary Table 6. *De novo* characterised *M. euphorbiae* genes related to biotransformation and detoxification.** Gene name, accession number, ORF and protein lengths of the *de novo* characterised *M. euphorbiae* genes as well as % of identity to closest species on databases.

| **Gene** | **Accession number** | **ORF length** | **Protein length** | **% identity** |
| --- | --- | --- | --- | --- |
| ***cyp4g15*** | MT105339 | 1701 bp | 566 aa | 99% (*Acyrthosiphon pisum*) |
| ***cyp6a13-like*** | MT105340 | 1122 bp | 373 aa (Incomplete) | 97% (*Acyrthosiphon pisum*) |
| ***cyp6k1-like*** | MT105341 | 299 bp | 99 aa (Incomplete) | 91% (*Acyrthosiphon pisum*) |
| ***cyp4c1*** | MT105342 | 630 bp | 209 aa (Incomplete) | 98% (*Acyrthosiphon pisum*) |
| ***Cu-Zn SOD-like*** | MT105343 | 465 bp | 154 aa | 63% (*Macconellicocus hirsutus*) |
| ***GST*** | MT105344 | 629 bp | 199 aa (Incomplete) | 76% (*Aedes aegypti*) |
| ***GPx*** | MT105345 | 454 bp | 150 aa (Incomplete) | *53%* (*Melanaphis sacchari*) |
